# Current News

**Published:** 1890-06

**Authors:** 


### Current News.

The St. Louis Dental Society, at a meeting held on April 1, passed the following resolution:

*Resolved*, That the St. Louis Dental Society favors holding the next meeting of the association at Excelsior, Mo., the original choice of its members.

The members, in an informal way, expressing a willingness that a change of date, to the week following the meeting of the Missouri State Dental Association, be made, our State meeting will be held on July 8, 9, 10, and 11.

The Society was also presented the "Call for an International Dental Congress" in 1892; it was laid on the table. It favors a memorial meeting of the American Dental Association in the year of the World's Fair.

John G. Harper,
*President, St. L. D. S.*

---

Chicago Dental Society.—At the annual meeting of the Chicago Dental Society, held on Tuesday, April 1, 1890, the following were elected officers for the ensuing year: President, C. N. Johnson; First Vice-President, C. H. Thayer; Second Vice-President, I. A. Freeman; Secretary, A. E. Baldwin; Corresponding Secretary, T. L. Gilmer; Treasurer, E. D. Swain; Librarian, A. W. Harlan; George H. Cushing to succeed himself on the Executive Committee; C. F. Hartt, E. A. Royce, and S. B. Palmer, Board of Censors.

T. L. Gilmer,
*Corresponding Secretary.*

---

Dental Society of the State of New York.—The following were elected officers, May 15, for this current year: President, W. W. Walker, New York City; Vice-President, G. L. Curtis, Syracuse, N. Y.; Treasurer, H. G. Mirick, Brooklyn, N. Y.; Secretary, F. T. Van Woert, Brooklyn, N. Y.; Correspondent, B. A. R. Ottolengui, Brooklyn, N. Y.

